# Non-Invasive Biomarkers for Assessing Liver Fibrosis in Biliary Atresia: A Literature Review

**DOI:** 10.3390/ijms27125295

**Published:** 2026-06-11

**Authors:** Gabriel Bența, Alina Grama, Alexandra Mititelu, Alexandru-Ștefan Niculae, Tudor Lucian Pop

**Affiliations:** 1 Second Pediatric Discipline, Department of Mother and Child, Iuliu Haţieganu University of Medicine and Pharmacy, 400177 Cluj-Napoca, Romania; benta.gabriel@elearn.umfcluj.ro (G.B.); tudor.pop@umfcluj.ro (T.L.P.); 2 Second Pediatric Clinic, Center of Expertise in Pediatric Rare Liver Diseases, Emergency Clinical Hospital for Children, 400177 Cluj-Napoca, Romania

**Keywords:** non-invasive biomarkers, fibrosis, biliary atresia

## Abstract

Biliary atresia (BA) is the leading indication for pediatric liver transplantation. In the absence of surgical treatment, BA progresses rapidly toward hepatic fibrosis and cirrhosis. Although liver biopsy remains the gold standard for histological evaluation, its utility is limited by invasiveness, associated risks, and sampling variability. These limitations have spurred the development and validation of noninvasive tools to evaluate liver fibrosis in this patient population. Multiple imaging techniques have been developed to assess liver fibrosis and cirrhosis. In recent years, additional BA-related biomarkers have been identified, showing significant potential for diagnosis, assessment of fibrosis severity, and prediction of native liver survival outcomes. This article reviews the roles and potential clinical applications of the following biomarkers: matrix metalloproteinase-7 (MMP-7), fibroblast growth factor 19 (FGF-19), interleukin-33 (IL-33), clusterin, and osteopontin. Further research is needed to confirm the utility of these prognostic biomarkers in predicting and improving outcomes in BA.

## 1. Introduction

Biliary atresia (BA) is recognized as the leading cause of infantile cholestasis and constitutes the principal indication for liver transplantation (LT) in children due to its relentless progression toward irreversible liver damage. Progressive injury of the extrahepatic bile ducts is a hallmark of BA and is associated with cholestasis, cholangiocyte hyperproliferation, and subsequent fibrotic and cirrhotic liver changes. It is a rare disease, with an incidence that varies geographically, ranging from approximately 1 in 10,000–19,000 live births in Europe and North America to higher rates of about 1 in 3000 live births in Asian countries [1,2,3,4].

The etiology of BA remains incompletely understood and is considered multifactorial, involving genetic predisposition, immune system abnormalities, and environmental or infectious triggers like Cytomegalovirus, Rotavirus, or Reovirus [4,5,6].

Infants with BA clinically present with persistent neonatal jaundice, acholic stools, dark urine, and hepatomegaly. Diagnosis is based on elevated direct bilirubin and cholestatic enzymes, supported by ultrasound findings such as gallbladder abnormalities and the triangular cord sign (echogenic fibrous tissue anterior to the portal vein), and confirmed by liver biopsy or intraoperative cholangiography. Early diagnosis of BA is essential, given the need for prompt surgical intervention. Kasai hepatoportoenterostomy (KPE) performed before 60 days—ideally before 30–45 days—significantly improves bile flow and prognosis, whereas delayed intervention (>90 days) is associated with poor outcomes and progression to cirrhosis [7,8].

In the absence of surgical treatment, BA progresses rapidly toward hepatic fibrosis and cirrhosis. Bile stasis leads to the accumulation of toxic bile acids, causing hepatocellular and cholangiocyte injury and triggering inflammatory and immune responses. Activation of hepatic stellate cells induced by these processes contributes to matrix accumulation and progressive alteration of liver architecture [4].

Global research on BA-related liver fibrosis has shown substantial growth over the past two decades [9]. Multiple imaging and blood-based methods have been developed to assess liver fibrosis and cirrhosis. In clinical practice, non-invasive imaging techniques are increasingly used to monitor fibrosis in chronic liver disease, including BA. Ultrasound-based elastography techniques, including transient elastography (TE) and shear wave elastography, together with magnetic resonance elastography (MRE), are among the methods currently used for assessment [9,10,11]. Recent advances have led to the identification of multiple serum biomarkers with potential diagnostic and prognostic applications in BA, many of which are now widely used, such as MMP-7 [12,13,14,15], IL-33 [16,17], FGF-19 [18,19], serum clusterin [20], total serum bile acids (SBA) [21], cartilage oligomeric matrix protein (COMP) [22], Mac-2 binding protein glycan isomer (M2BPGi) [23] and even Gamma glutamyl transpeptidase (GGT) [8,24,25]. Non-invasive tools to assess liver fibrosis are particularly attractive in BA, where serial biopsies are impractical and early risk stratification is crucial for preserving native liver function. Among serum-based indices, the AST-to-platelet ratio index (APRI) has emerged as a simple, widely available marker that correlates with fibrosis severity in BA, although variable cut-off values and heterogeneous study designs currently limit its use as a stand-alone decision tool [26].

**Figure 1:**
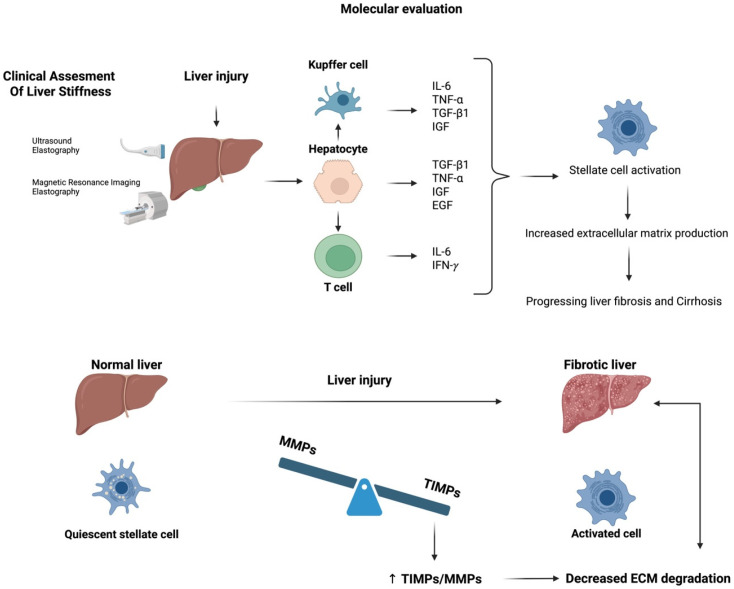
Non-invasive biomarkers of liver fibrosis based on the mechanisms of liver fibrogenesis. Liver injury triggers the release of inflammatory and profibrotic mediators from Kupffer cells, hepatocytes, and T cells, promoting hepatic stellate cell activation, increased extracellular matrix deposition, and fibrosis progression. An imbalance between matrix metalloproteinases and their inhibitors decreases extracellular matrix breakdown, contributing to fibrotic tissue buildup. Clinical assessment of liver stiffness includes ultrasound elastography and magnetic resonance imaging elastography. Abbreviations: ECM, extracellular matrix; MMPs, matrix metalloproteinases; TIMPs, tissue inhibitors of metalloproteinases; IL-6, interleukin-6; TNF-α, tumor necrosis factor alpha; TGF-β1, transforming growth factor beta 1; IGF, insulin-like growth factor; EGF, epidermal growth factor; IFN-γ, interferon gamma. Created in BioRender. Adam, S. (2026) https://BioRender.com/4gacrlb, accessed on 15 April 2026.

The aim of this narrative review is to evaluate the most important non-invasive biomarkers of liver fibrosis and their potential for further use in monitoring fibrosis in patients with BA (Figure 1). This narrative review was based on a literature search performed in PubMed and Scopus databases. Relevant English-language studies published between 2005 and 2026 were identified using combinations of the following keywords: “biliary atresia”, “liver fibrosis”, “non-invasive markers”, “serum biomarkers”, “transient elastography”, and “liver stiffness”.

## 2. Matrix Metalloproteinase-7

Matrix metalloproteinase-7 (MMP-7), commonly referred to as matrilysin-1, is an enzyme that contributes to extracellular matrix turnover and regulates tissue remodeling processes. It was discovered in 1988 by Sellers and Woessner as a rat uterine enzyme implicated in uterine changes during pregnancy, implantation, and postpartum uterine recovery [27]. MMP-7 is a secreted endopeptidase dependent on zinc and calcium ions, capable of degrading multiple components of the extracellular matrix (ECM), including elastin, fibronectin, type IV collagen, and laminin, thereby playing an essential role in tissue remodeling [28].

MMP-7 has a dual role: it regulates inflammation, wound healing, and bone development, and is a major contributor to the progression of various disorders such as cancer, renal disorders, liver, and pulmonary fibrosis [29]. It is a biomarker often overexpressed in various tumors (colorectal, pancreatic, lung, and breast cancers), facilitating cell proliferation, invasion, metastasis, and angiogenesis, and acting as a prognostic biomarker for poor outcomes. Recently, MMP-7 has been used as a new diagnostic test for cancer progression; its elevated levels indicated a more aggressive disease or a metastatic dissemination [30]. Human MMP-7 has a molecular weight of approximately 30 kDa and is encoded by the MMP7 gene, that have mapped to the region 11q21 → q22 [31].

MMP-7 is produced by various cells, especially epithelial cells, as an inactive precursor (zymogen) that is activated to degrade ECM components [32]. In the liver, MMP-7 acts as an inflammation promoter and is involved in bile cell proliferation or ECM degradation. This process results in liver injury, fibrosis, and tissue remodeling, which is often correlated with elevated levels of serum MMP-7 [33].

MMP-7 is a promising noninvasive biomarker for chronic liver disorders, reflecting the extent of injury and the liver’s remodeling capacity, through its ability to assess advanced fibrogenesis [34]. In metabolic-associated fatty liver disease (MAFLD), elevated serum levels of MMP-7 are associated with inflammation and liver fibrosis, predicting the risk of progression to more severe forms like metabolic-associated steatohepatitis (MASH) [35]. In autoimmune liver diseases (AILDs), such as autoimmune hepatitis (AIH) and primary sclerosing cholangitis (PSC), elevated MMP-7 levels are associated with bile duct injury, inflammation, and fibrosis, while also indicating a poorer prognosis [33]. In chronic hepatitis B and C, MMP-7 assesses liver inflammatory activity and predicts outcomes [36]. MMP-7 has a high expression in biliary epithelial cells, acting as a promising biomarker in infants with cholestatic disorders, being useful especially in differentiating BA from other causes of cholestasis [37]. Elevated levels of MMP-7 in BA contribute to the development of fibrosis, stimulate inflammatory pathways, and trigger apoptosis in biliary epithelial cells [38].

Lertudomphonwanit et al. [39] reported that cholangiocytes release MMP-7 in response to injury, as observed in an experimental mouse model of BA. Serum MMP-7 levels serve as indicators of liver fibrosis severity and may support the diagnosis of BA in cholestatic infants [40]. A serum MMP-7 concentration of 1.43 ng/mL predicted BA in infants with cholestasis, with a sensitivity of 97.30% and a specificity of 83.20%. A multicenter study [41] demonstrated higher sensitivity and specificity for MMP-7 compared to GGT (95.5% vs. 77.3% and 94.5% vs. 77.8%) in distinguishing BA from other causes of cholestasis. Also, elevated MMP-7 levels following KPE have been linked to an increased likelihood of LT in affected infants [40]. A significant correlation between serum MMP-7 values, age, and fibrosis stage was identified in patients with BA by Jiang et al., indicating that MMP-7 may contribute to fibrosis progression from early stages of the disease [13].

## 3. Fibroblast Growth Factor-19

Fibroblast Growth Factor 19 (FGF-19) has an essential role in metabolic regulation, particularly in maintaining bile acid balance as well as glucose and lipid metabolism. It is a key regulator of insulin and triglycerides, helping to prevent obesity and type 2 diabetes. It is a hormone-like protein mainly secreted by ileal enterocytes in the postprandial state, exerting its hepatic effects through fibroblast growth factor receptor 4 (FGFR-4) and the co-receptor β-klotho [42]. Increased serum levels of FGF-19 have been observed in cholestatic disorders, due to its expression in the gallbladder epithelium and hepatocytes [43]. The FGF-19 gene is located on chromosomal locus 11q13 and encodes a protein member of a family of FGFs, which is an important regulator of organogenesis during fetal development [44]. After binding FGF-19 to FGFR-4, this complex phosphorylates extracellular signal-regulated kinases (ERK) ERK1, ERK2, and the signal transducer and activator of transcription 3 (STAT-3), thereby influencing downstream target genes that regulate gluconeogenesis and lipogenesis.

The FGF-19-FGFR-4 axis also plays a vital role in regulating bile acid synthesis [45]. The synthesis of bile acids from cholesterol involves a cascade of enzymatic reactions regulated primarily by CYP7A1 (cholesterol 7α-hydroxylase), the rate-limiting enzyme of the process. FGF-19, produced in the ileum in response to bile acid absorption, enters the bloodstream and acts as an endocrine factor targeting the liver by binding to FGFR-4, which is highly expressed on hepatocytes [46,47]. Activation of ERK1 and ERK2 modulates transcription factors that suppress CYP7A1 gene expression, thereby decreasing bile acid synthesis. Concurrently, activated STAT-3 translocates to the nucleus and further represses CYP7A1 transcription [46,47]. This negative feedback loop effectively reduces the production of bile acids when their levels are sufficient, preventing their accumulation and potential cytotoxicity [46]. In liver diseases, disruption of the FGF-19-FGFR4 axis can have various consequences, such as impaired bile secretion, steatosis, fibrosis, or tumor development [48].

Several studies support that FGF-19 is an important biomarker in BA. Compared to age-matched controls, children with BA showed significantly elevated circulating FGF-19 levels [18]. Circulating FGF-19 levels in children with BA are of hepatic origin and are highly elevated prior to KPE, decreasing significantly after surgery [18,49]. Other researchers proved that serum FGF-19 levels remain elevated after KPE, particularly in children with ongoing cholestasis or poor bile drainage. These elevated FGF-19 levels are often associated with poorer outcomes after KPE, including a higher likelihood of requiring LT [50]. Zhu demonstrated that preoperative FGF-19 levels can predict native liver survival (NLS) in patients with early diagnosis (age at KPE ≤ 60 days). The same team supports the idea that postoperative FGF-19 can predict NLS after 1 year, with the persistence of elevated levels correlating with a poor prognosis [51]. In another multicenter cohort of 87 children with BA undergoing KPE, a FGF-19 level of 109 pg/mL at the time of surgery was correlated with an approximately four-fold risk of LT or death [19].

Elevated FGF-19 levels may help discriminate BA from alternative causes of neonatal cholestasis, supporting its role as a diagnostic biomarker. Significantly elevated levels of FGF-19 (223 vs. 61 pg/mL) were found in preoperative BA compared to other cholestatic etiologies, and may be useful in cases with an ambiguous diagnosis [19]. In BA, FGF-19 levels reflect chronic bile acid retention, inflammation, cholangiocyte injury, and liver fibrogenesis. Elevated FGF-19 levels reflect the body’s attempt to counteract bile acid overload, which drives hepatocyte injury and apoptosis, immune cell activation, cytokine release, bile duct proliferation, and stellate cell activation, collectively determining fibrosis. However, chronically high FGF-19 levels, particularly those originating from the liver, may directly contribute to fibrogenesis through FGFR-4 activation, which will stimulate mitogen-activated protein kinase (MAPK)/ERK signaling and will promote fibrosis-related genes [19,50,51]. Higher FGF-19 levels correlate with more severe liver fibrosis and worse outcomes after KPE, suggesting a prognostic role [19]. A positive correlation between circulating FGF-19 levels and model for end-stage liver disease (MELD) scores was observed by Zhanyi Li in individuals with cirrhosis related to primary biliary cholangitis–autoimmune hepatitis overlap syndrome (PBC-AIH) [52].

Another study from Poland focused on the implications of FGF-19 and FGF-21 (both belonging to the FGF-19 subfamily) in LT [53]. FGF-19 levels were significantly higher in patients with cirrhosis compared to healthy individuals. Also, there were significant changes in the plasma concentrations of the growth factors FGF-19 and FGF-21 before, at 24 h, and at 2 weeks after LT. The highest level of FGF-19 was observed before LT, a much lower level 24 h after this surgical intervention, and the lowest level was 2 weeks after surgery [53,54].

Serum concentrations of FGF-19 correlate positively with the severity of cholestasis, hepatocyte damage, and hepatic fibrosis. In addition, serum FGF-19 may help monitor carcinogenesis in adult patients. It acts as a driver oncogene in hepatocarcinoma (HCC), promoting tumor growth and spread by interacting with its receptor, FGFR-4, and activating downstream signaling pathways [47]. In patients with hepatocellular carcinoma (HCC), amplification of FGF-19 is commonly encountered and may contribute to future diagnostic and therapeutic applications [55].

## 4. Interleukin-33

Interleukin-33 (IL-33) belongs to the interleukin-1 (IL-1) family and plays important roles in tissue homeostasis, infection, inflammation, allergies, and numerous other diseases.

The structure of IL-33 includes 270 amino acids organized into a homeodomain-like helix–turn–helix (HTH) domain, a middle domain, and a C-terminal cytokine domain resembling IL-1 [56]. Like other IL-1 family cytokines, IL-33 is synthesized as a precursor (pro-IL-33). The mature molecule is formed by proteolysis, in which several molecules are involved, including calpain, elastase, and cathepsin G. The secretion of IL-33, both precursor and mature forms, is mediated by several pro-inflammatory cytokines such as TNF-α, IFN-γ, and IL-4 [57,58]. IL-33 fulfills its functions through a receptor complex, composed of IL-1 receptor-like 1 (IL1RL1, also known as ST2) and a co-receptor, IL-1 receptor accessory protein (IL1RAcP) [57,59].

IL-33 has multiple functions, both as a cytokine and as a transcriptional regulator [59]. The immunological activity of IL-33 involves activation of both Th2 and Th1 pathways, leading to the release of cytokines such as IL-4, IL-5, IL-13, IFN-γ, and TNF-α [57,60,61,62]. Additionally, IL-33 plays a crucial role in regulating the accumulation and effector functions of regulatory T cells, thereby contributing to immunosuppression and tissue repair. The process is mediated through direct or indirect stimulation of type 2 innate lymphoid cells (ILC2s) as well as macrophage polarization [57]. Moreover, defective IL-33 signaling is associated with various immune-related disorders, including allergies, asthma, rheumatoid arthritis, autoimmunity, organ fibrosis, and cardiovascular diseases [63].

IL-33 is continuously expressed in a wide variety of cell types, such as epithelial cells, endothelial cells, fibroblasts, cardiomyocytes, and certain immune cells, including macrophages, mast cells, and natural killer cells [64]. It can be found across multiple organs, including lymphoid tissues, the brain, lungs, embryos, epithelial barrier tissues, and inflamed tissues [56,65].

A broad spectrum of lymphoid and myeloid immune cells expressing the ST2 receptor can be activated by IL-33 released from necrotic cells. Its functions can vary dramatically depending on the disease context; it may promote inflammation in some instances while supporting immunosuppression in others [66].

In the liver, stressed hepatocytes release IL-33 as an alarmin, drawing ILC2s to the organ. When cultured hepatic stellate cells (HSC) are stimulated with IL-33, they express fibrogenic cytokines such as IL-6 and TGF-β, along with markers of hepatic fibrogenesis like α-smooth muscle actin (α-SMA) and collagen, in activated and transdifferentiated HSCs, also referred to as myofibroblasts (MFB) [67]. Activation of hepatic stellate cells, the major producers of extracellular matrix in the liver, is strongly influenced by the IL-33/ST2 signaling pathway. Treatment of these culture-activated cells with recombinant IL-33 enhances activation of the MAPK pathway via ERK, c-Jun N-terminal Kinase (JNK), and p38, leading to increased expression of α-SMA and collagen. These findings indicate a direct profibrogenic role of IL-33 in HSCs, which may work synergistically with its influence on ILC2 [67,68].

The involvement of IL-33 has been demonstrated in studies of mice with bile duct ligation-induced fibrosis and in humans with cirrhosis [68]. The importance of IL-33 in liver fibrosis is also supported by studies demonstrating improvements in fibrosis after inhibiting the IL-33 signaling pathway [69].

Damage to the biliary epithelial lining triggers inflammation and fibrosis, which can present clinically as severe inflammatory diseases in children (such as BA) and in adults (such as sclerosing cholangitis). Despite the severity of the disease presentation, the cellular and molecular mediators underlying bile duct regeneration and their contributions to carcinogenic pathways remain insufficiently elucidated. Analyses of BA pathogenesis, the most common end-stage cholangiopathy of childhood, have shown that affected livers contain lymphocytes at diagnosis. Recent investigations have shown that, despite the predominance of a type 1 immune response in most patients with BA at diagnosis, some individuals display elevated Th2 cytokine levels along with increased hepatic expression of IL1RL1, the gene responsible for encoding the IL-33 receptor ST2 [70].

These findings have been validated by recent studies showing elevated levels of IL-33 in BA patients compared to those with cholestatic causes or healthy controls. Li demonstrated higher levels of IL-33 in patients with an unfavorable prognosis after KPE compared to those with good outcomes [70]. Behairy et al. reported that BA patients had significantly higher IL-33 levels than those with other cholestatic causes, and both groups had higher serum IL-33 levels than the control group. They concluded that a value over 20.8 ng/mL can detect BA with a specificity of 95% and a sensitivity of 96.7% [17]. These studies highlight a noninvasive marker that could be used for early diagnosis, prognostication of severe forms, or even for new treatment options, given IL-33’s role in the development of liver fibrosis.

## 5. Clusterin

Clusterin (CLU) is an extracellular chaperone present intracellularly and extracellularly in almost all body fluids. CLU is a glycoprotein present in various tissues and body fluids. It is involved in multiple biological processes, including cell differentiation, apoptosis, immune regulation, and lipid transport [71,72]. It has a dual role, acting to promote cellular protection against stress-induced apoptosis and, in some contexts, can regulate programmed cell death [71]. CLU is secreted intracellularly, especially by the specialized secretory and epithelial cells, but also by hepatocytes and cholangiocytes [73].

CLU exerts beneficial effects in cholestatic liver diseases by reducing liver inflammation and fibrosis. CLU suppresses the activation and proliferation of hepatic stellate cells (HSCs), thereby decreasing collagen production. Increased serum CLU levels observed in patients with cholestatic liver disorders function as a protective mechanism by inhibiting hepatocyte apoptosis and alleviating cellular stress and inflammation [74,75]. But in severe forms of cholestatic disorders, which have already progressed to fibrosis, CLU is an active mediator for liver fibrogenesis [75,76]. In animal models of liver injury, it has been observed that in the early stages, CLU expression increases, correlating with a favorable evolution [75,76]. CLU overexpression in response to chronic liver injury is associated with the accumulation of elastic fibers, being a marker of unfavorable evolution [76].

In BA, circulating CLU levels might reflect the disease progression after KPE. According to Udomsinprasert et al., lower serum CLU concentrations correlated with poorer survival following KPE in BA patients, especially among individuals with persistent jaundice, severe fibrosis, and advanced hepatic dysfunction [20].

## 6. Osteopontin

Biomarkers such as osteopontin (OPN), hyaluronic acid, and procollagen peptides are also being explored as tools for fibrosis assessment and disease staging [77,78,79,80]. OPN is a multifunctional glycoprotein that plays a significant role in immune responses, inflammation, tissue repair, and fibrosis. OPN is involved in immune cell recruitment and the production of proinflammatory cytokines, contributing to chronic inflammation and tissue damage. At the hepatic level, they cause the accumulation of ECM components, promoting fibrosis [79]. It is an important marker of liver fibrosis progression, being considered comparable or superior to other non-invasive fibrosis assessment markers or methods (like TE, FibroScan), especially in distinguishing advanced fibrosis or cirrhosis [81]. In BA, serum OPN may predict disease progression, degree of fibrosis, and post-KPE outcome. In a study by Aldeiri et al., serum OPN was significantly higher in BA patients compared with controls (median 1952 vs. 1457 ng/mL). It demonstrated moderate diagnostic performance at a cutoff of 1611 ng/mL, yielding 84% sensitivity and 78% specificity (NPV 81%); however, unlike MMP-7, OPN levels did not correlate with Ishak fibrosis stage and were not predictive of jaundice clearance or the need for LT [78]. In BA patients, OPN was found in significant quantities in interlobular biliary epithelium, correlating with biliary proliferation and portal fibrosis. This is not found in other cases of cholestasis or in the healthy liver [80]. Other authors rightly suggest that OPN is implicated in the pathogenesis of BA in humans [82]. Hertel et al. used immunoperoxidase staining for OPN and Cytokeratin-19 (CK-19) (a bile duct epithelial marker) and demonstrated intense OPN positivity in bile duct-associated areas in healthy murine livers as well as in those with BA and reovirus infection [83]. They concluded that OPN is expressed in the intra- and extra-hepatic bile ducts of normal mice as well as in the bile ducts of mice with BA, but in the latter, it is found in much greater quantities as a marker of inflammation and/or fibrogenesis [83].

## 7. Imaging Biomarkers

Beyond molecular biomarkers, imaging methods—especially ultrasound-based elastography techniques—have become increasingly important in evaluating disease progression and forecasting outcomes in BA following KPE.

TE is a noninvasive ultrasound-based technique using high–frame rate shear waves to quantify liver stiffness and stage liver fibrosis in adults and children [84]. Yeung et al. found that long-term survivors of BA with a native liver had a median TE liver stiffness score of 11.4 kPa, and higher stiffness values were significantly associated with complications of portal hypertension, including esophageal varices, supporting TE (FibroScan^®^) as a useful non-invasive screening tool [85]. Another study by Shin et al. supports that TE showed a strong correlation with METAVIR fibrosis stages and demonstrated excellent accuracy for detecting severe fibrosis (AUC 0.86; cutoff > 9.6 kPa; sensitivity 89.5%, specificity 75%) and cirrhosis (AUC 0.96; cutoff > 18.1 kPa; sensitivity 100%, specificity 90.5%) in infants with BA, supporting its role as a reliable noninvasive tool to guide preoperative assessment and reduce the need for invasive liver biopsy [86]. Honsawek et al. found that TE-derived liver stiffness correlated with the degree of hepatic fibrosis in children with BA. In the same study, serum adiponectin levels were also associated with liver stiffness, indicating a potential complementary role for biochemical markers alongside imaging-based fibrosis assessment [87].

Shear wave elastography (SWE), a quantitative ultrasound modality, has demonstrated potential for detecting advanced liver fibrosis both before and after KPE [88]. According to Yoon et al., pre-KPE liver SWE (cutoff 17.5 kPa) accurately predicts advanced fibrosis (F3–F4) with 86% specificity, while postoperative SWE on day 3 (cutoff 10.3 kPa) perfectly identifies patients with poor outcomes (100% sensitivity) after the Kasai procedure [89]. Another study performed by Ahmad et al. showed that shear wave elastography (SWE) was shown to be a reliable noninvasive alternative to liver biopsy for evaluating fibrosis in BA. SWE measurements correlated strongly with liver fibrosis severity (*p* < 0.001). At 3 months after KPE, children with successful bile drainage had lower liver stiffness (12.8 kPa vs. 17.3 kPa) (*p* < 0.001). These findings suggest that SWE may help predict Kasai outcomes and monitor fibrosis noninvasively [90]. Sekarsari et al. showed that SWE has significant prognostic value for predicting post-KPE outcomes in BA, with an optimal cutoff of 2.21 m/s (14.4 kPa) yielding 88.9% sensitivity and 83.3% specificity [91].

MRE is a highly accurate non-invasive alternative to liver biopsy for staging fibrosis in children that can reliably identify and stage fibrosis in children [92]. Kim et al. demonstrated that 3T spin-echo echo-planar imaging (SE-EPI) MRE is technically feasible in children and young adults with chronic liver disease, including patients with BA after KPE. Liver stiffness measurements obtained with SE-EPI MRE were higher in BA patients compared with patients undergoing MRI for fatty liver evaluation, supporting the role of MRE as a non-invasive biomarker of hepatic fibrosis [93]. Spleen MRE was shown to be a useful noninvasive marker of portal hypertension and gastroesophageal varices in children with BA after KPE. A spleen stiffness cutoff of 9.9 kPa predicted the presence of varices with an AUC of 0.844, similar to APRI and spleen size ratio, whereas liver MRE values were not discriminatory [94].

## 8. Proposed Use of Non-Invasive Biomarkers in the Monitoring of Biliary Atresia Patients

Noninvasive biomarkers have the potential to improve the monitoring of children with BA throughout the entire clinical course—from diagnosis and the pre-KPE period, through the post-operative phase—by providing serial, objective assessments of native-liver fibrosis and functional reserve. By integrating conventional liver-function tests with serological fibrosis indices (such as APRI and FIB-4) and elastography, clinicians might decrease dependence on repeated liver biopsies while improving early detection of progressive fibrosis and portal hypertension. According to Brujats et al., elastography-based techniques have become increasingly important for the longitudinal evaluation of graft fibrosis and portal hypertension, potentially reducing reliance on repeated liver biopsies in transplant recipients [95].

In the post-Kasai setting, serial biomarker evaluation could help stratify risk for transplant-free survival, guide timely referral for LT, and support individualized follow-up strategies, ultimately contributing to better long-term outcomes for children with BA (Table 1).

BA requires improved monitoring strategies, given the heterogeneous long-term outcomes following KPE and the current reliance on bilirubin levels, imaging, clinical evaluation, and invasive or semi-invasive assessments for follow-up. Recent reviews and studies underscore the potential of biomarkers primarily for prognostic evaluation and risk stratification, beyond their established diagnostic utility.

Biomarkers in BA should ideally provide a more comprehensive assessment of disease progression by tracking bile drainage efficacy and residual cholestasis, fibrosis progression and cirrhosis risk, portal hypertension and the development of high-risk varices, as well as cholangitis detection and broader inflammatory activity. In addition, they should help predict NLS and transplant-free survival, allowing for earlier risk stratification, more individualized follow-up, and better identification of children who may benefit from closer surveillance or timely escalation of care [96,97,98].

A practical monitoring plan for BA should begin with a baseline assessment soon after KPE, followed by serial measurements over time rather than isolated testing. This approach should integrate biomarkers with patient age, clinical findings, and elastography to improve overall risk assessment. It should also place greater emphasis on biomarker trajectories over time rather than fixed thresholds alone, as this may allow earlier recognition of deterioration and more timely clinical intervention. In this way, monitoring would be better aligned with the dynamic nature of BA and the heterogeneous course observed after surgery [98,99].

**Table 1:** Summarization of the evidence regarding the potential roles of novel serum biomarkers in biliary atresia (BA).

Biomarker	Role/Utility	Description	Reference
MMP-7	Diagnosis of BA	MMP-7 demonstrated strong discriminatory power for BA across two validation cohorts, achieving 95% diagnostic accuracy when combined with GGT.	[39]
		Serum MMP-7 levels, reflecting liver fibrosis severity noninvasively, aid BA diagnosis in cholestatic infants.	[40]
		MMP-7 showed 95.5% sensitivity and 94.5% specificity for distinguishing BA from non-BA cholestasis.	[41]
		Diagnostic marker for BA differentiation from other cholestatic diseases.	[100]
		Serum MMP-7 exhibits strong discriminatory ability to distinguish BA from other neonatal cholestasis forms, though cutoff values differ by assay method.	[101]
	Liver fibrosis	MMP-7 levels significantly correlate with the stage of liver fibrosis in BA patients (R = 0.47; *p* < 0.001).	[13]
		MMP-7 is upregulated in biliary epithelium and serum post-KPE in BA, positively correlating with liver fibrosis stage (r = 0.605, *p* = 0.001) and portal fibrosis, suggesting its essential role in progressive fibrogenesis.	[102]
		MMP-7, endoglin, and IL-8 show significant correlations with elevated liver stiffness in children with BA.	[38]
	Predicting NLS after KPE	Serum MMP-7 was the sole significant predictor of 2-year NLS at 6 weeks post-KPE and the most accurate at 3 months post-KPE.	[101]
FGF-19	Predicting NLS after KPE	Elevated serum FGF19 at KPE predicted poorer long-term NLS in BA, correlating with unsuccessful KPE, higher primary bile acids, and ductular reaction. Serum FGF19 is elevated in BA (223 vs. 61 pg/mL in controls) and predicts poorer native liver survival, with levels > 10^9^ pg/mL associated with a 4.31-fold higher hazard of liver loss, and is higher in transplant patients (410 vs. 99 pg/mL).	[19]
		Postoperative FGF-19 can predict NLS after 1 year, with elevated levels correlating with a poor prognosis.	[51]
IL-33	Diagnosis of BA	Serum IL-33 at a cut-off value of 20.8 pg/mL is a highly accurate diagnostic biomarker for BA in infants with neonatal cholestasis, with 96.7% sensitivity and 95% specificity, and its levels positively correlate with elevations in liver enzymes and fibrosis severity.	[17]
		Serum and liver/bile duct IL-33, a Th2-activating cytokine, is elevated in BA patients and experimental mouse models.	[70]
Clusterin	Predicting NLS after KPE	CLU may indicate poor outcomes in BA patients and shows promise as a novel biomarker for disease severity after KPE. CLU has an AUC of 0.85, with 81.5% sensitivity and 73.5% specificity, for predicting poor outcomes in BA patients after a Kasai operation.	[20]
Osteopontin	Liver fibrosis	In BA patients, osteopontin was found in significant quantities in biliary epithelium, correlating with biliary proliferation and portal fibrosis. ROC analysis identified an OPN cut-off of ≥1611 ng/mL for detecting BA, with 84% sensitivity, 78% specificity, 82% PPV, and 81% NPV.	[78]
		Osteopontin is localized in interlobular biliary epithelium, correlating with biliary proliferation and portal fibrosis in BA patients.	[80]
		Elevated circulating osteopontin correlates with hepatic dysfunction and portal hypertension in BA patients, serving as a potential marker for disease severity and progression monitoring post-Kasai.	[103]

Clinical applications include identifying children with adequate bile drainage but at risk of persistent fibrosis, detecting patients at risk of portal hypertension before decompensation, informing the timing of endoscopic surveillance, aiding stratification for LT referral, and selecting homogeneous cohorts for future therapeutic trials.

Routine measurement of accurate and validated biomarkers as part of clinical BA research would contribute significantly to a deeper understanding of liver fibrosis in this patient population [11]. These biomarkers are not yet standard of care due to small cohorts and single-center studies, variability in assays and cutoffs across studies, limited external validation, age dependence of several markers, and the absence of prospective studies demonstrating improved outcomes with biomarker-guided follow-up [98,104].

Despite its promising diagnostic accuracy for BA, clinical implementation of serum MMP-7 faces critical challenges, including a lack of assay standardization across platforms, the need for age-specific cutoffs—particularly higher thresholds in neonates—and the necessity for multicenter external validation in diverse populations before routine adoption [105]. Although serum FGF-19 demonstrates prognostic value for predicting NLS after KPE, its clinical implementation is hindered by a lack of assay standardization across ELISA platforms, the absence of age-specific cutoffs despite conflicting reports on whether low (<10^9^ pg/mL) or high levels predict poor outcomes, and the critical need for multicenter external validation in larger, diverse cohorts before routine prognostic use [19]. Although serum IL-33 demonstrates exceptional diagnostic accuracy for BA (AUC 0.995, sensitivity 96.7%, specificity 95% at cutoff ≥ 20.8 pg/mL), clinical implementation is hindered by a lack of assay standardization across ELISA platforms, the absence of age-specific cutoffs despite limited age-range inclusion, and the critical need for multicenter external validation in diverse populations before routine clinical adoption [12].

Beyond established fibrosis-associated biomarkers, apoptosis-related markers have also been explored. Serum caspase-3 activity was reported to differentiate BA from other cholestatic disorders with high sensitivity and specificity, suggesting potential utility as a noninvasive indicator of disease activity [106]. However, evidence remains limited and requires validation in larger cohorts.

Future research priorities for prognostic biomarkers in BA emphasize multicenter longitudinal validation to overcome limitations of small, single-center cohorts. The field has increasingly shifted toward composite biomarker scores and serial monitoring rather than relying on single biomarkers.

Recent evidence demonstrates that combining MMP-7 with GGT and bile acids significantly improves diagnostic accuracy: an integrated model of MMP-7 + Tauroursodeoxycholic acid (TUDCA) + Glycoursodeoxycholic acid (GUDCA)/Ursodeoxycholic acid (UDCA) ratio achieved AUC = 0.976, while the full model including MMP-7 + GGT + bile acids reached AUC = 0.983 (sensitivity 93%, specificity 98.3%) [107]. Composite scores integrating serum markers (such as bile acids, MMP-7, FGF-19) with noninvasive imaging modalities, such as elastography, could enhance the accuracy of predicting fibrosis progression and NLS post-KPE. Beyond composite scores, serial monitoring of biomarkers provides critical prognostic information. Dynamic analysis of MMP-7 from pre-KPE to post-KPE reveals four distinct patterns that predict 2-year native liver survival, with the most accurate prediction at 3 months post-KPE [100].

Standardization of assay platforms and cutoffs is essential for reducing variability across studies and enabling reliable clinical translation. Prediction models targeting portal hypertension, recurrent cholangitis, and transplant-free survival are gaining traction, with emerging data supporting serial biomarker monitoring to capture disease trajectories rather than static snapshots. Platelet count <100 × 10^9^/L and the varices prediction rule (VPR = [albumin × platelet count]/1000) with a cutoff ≤ 3.0 also demonstrated superior performance (AUROC 0.83, sensitivity 81.3%, specificity 85.7%, NPV 94.1%) for predicting portal hypertension complications compared to individual markers alone [108]. Yoneyama et al. reported that hepatic overexpression of miR-214 is associated with progression of liver fibrosis in patients with BA, with significantly elevated circulating miR-214 levels in patients with severe fibrosis [109].

Future approaches may combine serum biomarkers, elastography parameters, and artificial intelligence-assisted imaging analyses to improve fibrosis prediction. Such multimodal strategies are already being proposed for chronic liver diseases and may ultimately enhance fibrosis monitoring in BA [110].

Finally, the integration of omics approaches—such as hepatic gene expression signatures, circulating microRNAs, and immune activation markers—with routine follow-up tools holds promise for precision risk stratification and selecting homogeneous patient groups for therapeutic trials [88].

## 9. Limitations

Future biomarker implementation for BA faces several shared challenges across MMP-7, FGF-19, IL-33, OPN, and CLU. Small cohorts dominate the literature, with most studies including fewer than 100 BA patients. Most studies include single-center designs without multicenter validation in diverse populations. Assay heterogeneity creates substantial variability. Lack of prospective validation is critical. The CLU evidence base relies almost exclusively on a single research group (Thailand) [20]. No independent replication exists from other institutions or geographic regions, making this a critical gap in the evidence hierarchy. Until independent groups validate these findings, clusterin’s prognostic utility for post-Kasai outcomes remains preliminary.

## 10. Conclusions

Noninvasive biomarkers may substantially refine the management of BA by enabling earlier diagnosis, reducing the need for invasive diagnostic tests, improving prognostic stratification, and facilitating more individualized therapeutic strategies, all of which are critical for optimizing NLS in affected infants. Further research is necessary to confirm the effectiveness of these prognostic biomarkers in predicting outcomes and guiding improved management in BA.

## Data Availability

No new data were created or analyzed in this study. Data sharing is not applicable to this article.

## Abbreviations

The following abbreviations are used in this manuscript:

BA	Biliary atresia
MMP-7	Matrix metalloproteinase-7
FGF-19	Fibroblast growth factor 19
IL-33	Interleukin-33
LT	Liver transplantation
KPE	Kasai hepatoportoenterostomy
TE	Transient elastography
MRE	Magnetic resonance elastography
SBA	Serum bile acids
COMP	Cartilage oligomeric matrix protein
M2BPGi	Mac-2 binding protein glycan isomer
GGT	Gamma-glutamyl transpeptidase
APRI	Aspartate aminotransferase to platelet ratio index
ECM	Extracellular matrix
TIMP	Tissue inhibitors of metalloproteinases
IL-6	Interleukin-6
TNF-α	Tumor necrosis factor alpha
TGF-β1	Transforming growth factor beta 1
IGF	Insulin-like growth factor
EGF	Epidermal growth factor
IFN-γ	Interferon gamma
MAFLD	Metabolic-associated fatty liver disease
MASH	Metabolic-associated steatohepatitis
AILD	Autoimmune liver disorders
AIH	Autoimmune hepatitis
PSC	Primary sclerosing cholangitis
FGFR-4	Fibroblast growth factor receptor 4
FGF	Fibroblast growth factors
ERK	Extracellular signal-regulated kinases
STAT-3	Signal transducer and activator of transcription-3
CYP7A1	Cholesterol 7 alpha-hydroxylase
NLS	Native liver survival
MAPK	Mitogen-activated protein kinase
MELD	Model for end-stage liver disease
PBC-AIH	Primary biliary cholangitis-autoimmune hepatitis
HCC	Hepatocarcinoma
HTH	Helix-turn-helix
IL1RL1	Interleukin-1 receptor-like 1
ST2	Growth stimulation-expressed gene 2
IL1RacP	Interleukin-1 receptor accessory protein
Th1	Type 1 helper
Th2	Type 2 helper
ILC	Innate lymphoid cells
HSC	Hepatic stellate cells
α-SMA	alpha-smooth muscle actin
MFB	Myofibroblasts
JNK	c-Jun N-terminal Kinase
CLU	Clusterin
OPN	Osteopontin
CK-19	Cytokeratin-19
SWE	Shear wave elastography
SE-EPI	spin-echo echo-planar imaging
TUDCA	Tauroursodeoxycholic acid
GUDCA	Glycoursodeoxycholic acid
UDCA	Ursodeoxycholic acid
FIB-4	Fibrosis-4 index
